# COVID-19 Public Health Communication on X (Formerly Twitter): Cross-Sectional Study of Message Type, Sentiment, and Source

**DOI:** 10.2196/59687

**Published:** 2025-03-19

**Authors:** Sana Parveen, Agustin Garcia Pereira, Nathaly Garzon-Orjuela, Patricia McHugh, Aswathi Surendran, Heike Vornhagen, Akke Vellinga

**Affiliations:** 1 School of Public Health, Physiotherapy and Sports Science, University College Dublin, Belfield, Dublin 4, Ireland, Dublin, D04 V1W8, Ireland, 353 017163445; 2 Data Science Institute, University of Galway, Galway, Ireland; 3 J.E. Cairnes School of Business & Economics, University of Galway, Galway, Ireland, Ireland; 4 School of Medicine, Ollscoil na Gaillimhe – University of Galway, Galway, Ireland

**Keywords:** public health communication, surveillance, COVID-19, SARS-CoV-2, coronavirus, respiratory, infectious, pulmonary, pandemic, public health messaging, healthcare information, social media, tweets, text mining, data mining, social marketing, infoveillance, intervention planning

## Abstract

**Background:**

Social media can be used to quickly disseminate focused public health messages, increasing message reach and interaction with the public. Social media can also be an indicator of people’s emotions and concerns. Social media data text mining can be used for disease forecasting and understanding public awareness of health-related concerns. Limited studies explore the impact of type, sentiment and source of tweets on engagement. Thus, it is crucial to research how the general public reacts to various kinds of messages from different sources.

**Objective:**

The objective of this paper was to determine the association between message type, user (source) and sentiment of tweets and public engagement during the COVID-19 pandemic.

**Methods:**

For this study, 867,485 tweets were extracted from January 1, 2020 to March 31, 2022 from Ireland and the United Kingdom. A 4-step analytical process was undertaken, encompassing sentiment analysis, bio-classification (user), message classification and statistical analysis. A combination of manual content analysis with abductive coding and machine learning models were used to categorize sentiment, user category and message type for every tweet. A zero-inflated negative binomial model was applied to explore the most engaging content mix.

**Results:**

Our analysis resulted in 12 user categories, 6 message categories, and 3 sentiment classes. Personal stories and positive messages have the most engagement, even though not for every user group; known persons and influencers have the most engagement with humorous tweets. Health professionals receive more engagement with advocacy, personal stories/statements and humor-based tweets. Health institutes observe higher engagement with advocacy, personal stories/statements, and tweets with a positive sentiment. Personal stories/statements are not the most often tweeted category (22%) but have the highest engagement (27%). Messages centered on shock/disgust/fear-based (32%) have a 21% engagement. The frequency of informative/educational communications is high (33%) and their engagement is 16%. Advocacy message (8%) receive 9% engagement. Humor and opportunistic messages have engagements of 4% and 0.5% and low frequenciesof 5% and 1%, respectively. This study suggests the optimum mix of message type and sentiment that each user category should use to get more engagement.

**Conclusions:**

This study provides comprehensive insight into Twitter (rebranded as X in 2023) users’ responses toward various message type and sources. Our study shows that audience engages with personal stories and positive messages the most. Our findings provide valuable guidance for social media-based public health campaigns in developing messages for maximum engagement.

## Introduction

Public health communication is the scientific research, strategic transmission, and critical evaluation of health information to promote public health [1]. Public health communication initiatives can result in change by increasing awareness, boosting knowledge and forming attitudes when initiatives are well-planned, meticulously carried out, and sustained over time [2].

Social media can be used to quickly disseminate focused public health messages, increasing message reach and interaction with the general public [3,4]. Identifying and understanding information needs, false information, hate speech and discrimination, adherence to precautions, and where concerns lay, aids in the customization of public health strategy and eventually, the development of more informed interventions [5].

Social media can be a valuable resource for learning about people’s emotions, concerns and exchanging information. This was shown for instance, when Ebola broke out in Nigeria and public health institutions assisted in containing the Ebola outbreak by tracking social media interactions and disseminating accurate information about the illness [6]. Social media allows public health institutions to track outbreaks in real time and Twitter (rebranded as X in 2023) has been frequently used as a communication tool [7]. The features and status of disease outbreaks can be predicted and explained using information from social media sites and user-generated information has supported the development of early response methods [8].

Social media data text mining can be used for disease forecasting and understanding public awareness of health-related concerns [8]. However, it is still unclear how different social media messages are shared and interpreted or whether different sources (individuals or institutions) communicate efficiently.

During the COVID-19 pandemic [9], social media successfully informed and increased public awareness about this new phenomenon [10]. However, there were considerable differences in the preferred social media platforms, message formats and source sender types [11].

An important concern during the COVID-19 pandemic was the spread of misinformation on social media [12]. Research shows that promoting more messages from reputable, authoritative sources on social media is one of the best ways to prevent misinformation [13].

Examining the content of social media messages provides valuable and timely insights regarding public awareness levels and their needs [14]. While there have been many studies analyzing the tweets on various health issues, there has been limited studies to explore the impact of type, sentiment and source of posts on engagement in public health communication. This study used tweets sent during the pandemic to explore 3 research questions:

- Which sources of information are effective in public health communication?
- What are the most effective types of messages in public health communication?
- Which message type should different sources use to improve engagement?

## Methods

### Data Collection

Data were extracted from Twitter using a Python script to communicate with Twitter Rest API (application programming interface) using the “Search” endpoint. The “query” parameter was used to filter the results based on the “has:geo” tag, the “place_country:UK”/ “place_country:IE” tag (for Ireland and the United Kingdom), and “lang:en” tag (for English). The ”start_time” and “end_time” parameters were used to filter the posts from January 1, 2020 to March 31, 2022.

A basic search was conducted with phrases such as “coronavirus,” “SARS-CoV-2,” and “COVID-19” on Twitter. Using an iterative method, keywords were added and removed in order to find the most suitable for the data search. The final list of 10 keywords for data extraction included “COVID-19,” “pandemic,” “SARS-CoV-2,” “coronavirus,” “SARS-CoV-2 virus,” “social distancing,” “self-isolation,” “self-quarantine,” “quarantine,” and “new variant.” The total number of tweets extracted totaled 867,485.

### Data Analysis

Due to the extensive volume of data, a systematic approach was adopted using a 4-step analytical process including sentiment analysis, user-classification, message classification and statistical analysis (Table 1). A combination of manual coding and machine learning (ML) models were used for sentiment, user and message classification. This allowed for multiple rounds of coding to increase the robustness of our results. This methodology is also used by Kummervold et al in their study which shows that by utilizing machine learning models, they could almost exactly match the accuracy of a single human coder when it came to tweet classification. Their research indicates that this automated method, which is dependable and accurate, may be able to guide potentially useful and essential interventions while also freeing up important time and resources for carrying out similar analyses [15].

**Table 1.** Phase wise description of the data analysis process.

Phase	Tasks	Methods	Outcome
Data collection	Identification of keywords related to COVID-19	Identification of 15 keywords. After discussion among research team, reduced to 10 keywords for data extraction.	Final keywords
	Data extraction	An academic researcher access was applied for with Twitter. 867,485 tweets extracted.	
Sentiment analysis	Allocate sentiment to each tweet	Random selection of 260 tweets for sentiment allocation (positive, negative, and neutral). A total of 3 coders manually assigned sentiments to 1 set of 260 random tweets. Using majority voting, out of 260 tweets, coders agreed on 247 tweets (2 or 3 coders voted the same). Kappa statistic calculated between all coders and the models. RoBERTa-base model (highest agreement) applied to the whole dataset.	Manual coded tweets Kappa statistic results Tweets sentiment assigned—full dataset
User classification	Identify user categories	User categories identified based on literature on social media supported public health interventions. Definition of 5 user categories: health institute, health professional, influencer, researcher, and public.	User categories
	Coding user profiles	Abductive coding by 4 Manual coders on 1000 randomly selected tweets to identify new user categories. After 3 rounds of manual coding, most discrepancies or disagreements resolved through discussion. Using majority voting, out of 250 tweets, coders agreed on 165 tweets (at least 3 coders voted the same). A total of 3 ML models selected to assign user categories to same set of 250 tweets used in Round 3 of manual coding. Bag of words were defined for each user category for user categorization by the ML models. Accuracy score and *F*_1_-score calculated between ML models and manual coders. SetFit model (highest agreement) applied to the whole dataset.	User classification coding—Round 1, 2, and 3 Bag of words Accuracy and *F*_1_-score Tweets users assigned—full dataset
Message classification	Identify message categories	Message categories defined based on review of public health communication literature. Definition of 5 message categories: humor, shock/disgust, informative/educational, opportunistic, and personal stories.	Message categories
	Coding tweets	Abductive coding by 5 manual coders on randomly selected tweets in 3 rounds: 100 tweets in round 1, 100 tweets in round 2, and 250 tweets in round 3. After 2 rounds of manual coding, discrepancies or disagreements resolved through discussion. A total of 6 final message categories after addition and reduction of categories over 3 rounds. Using majority voting, out of 250 tweets, coders agreed on 203 tweets (3 or more coders voted the same). GPT-3 and Setfit model to assign message categories to set of 250 tweets from the final round using 6 message categories. Accuracy score and *F*_1_-score calculated between models and manual coder. SetFit model (highest agreement) applied to the whole dataset.	Manual coding—Round 1, 2, and 3 Tweets message assigned—Tweet users assigned
Statistical analysis	Engagement calculation	Exclusion of zero-follower users (n=537). Calculation of engagement (sum of likes, replies, retweets and quoted tweet count divided by the respective user’s followers)	
	Zero-inflation model	Exclusion of public user group (outside of objective of research) due to 87% zero-engagement. Application of zero-inflated Poisson and zero-inflated negative binomial model due to remainder of 26% zero-engagement. Selection of zero-inflated negative binomial model (best fit) with informative/educational message type and neutral sentiment as comparator.	Final model results

Our cross-sectional observational study aims to provide guidance for social media-based public health campaigns in developing messages for maximum engagement. The study adhered to the Strengthening the Reporting of Observational Studies in Epidemiology (STROBE) checklist for cross-sectional studies (Checklist 1). Public health interventions are mostly run by governmental agencies or health institutes but can also involve collaborations with researchers, influencers, artists, etc. Therefore, it is important to study how the public engages with different types of messages from various sources. To ensure a clear distinction and understanding of public engagement, tweets by the general public were removed from our final analysis to focus on engagement received by tweets from all other sources.

#### Sentiment Analysis

A subset of 260 tweets was randomly extracted for manual annotation by three coders with sentiment labels - positive, negative or neutral (Multimedia Appendix 1). Subsequently, we used 3 ML sentiment analysis models to detect the sentiment for the same tweet subset, which were: distilbert-base-uncased-finetuned-sst-2-english [16], finite automata/bertweet-base-sentiment-analysis [17], and RoBERTa-base for Sentiment Analysis [18].

The kappa statistic was calculated between the 3 coders and between each coder and model. The model with the highest agreement with manual coders based on accuracy score [19] and *F*_1_-score [20] was applied to the full dataset.

#### User Classification

A directed content analysis with abductive coding was used to explore which sources of information are effective in public health communication. User categorization was based on the profile description and categorized according to an adaptation of the user categories from Cole-Lewis [21]: health institute, health professional, influencer, researcher and public.

User coding was performed in 3 cycles. The profile description of 1000 randomly selected tweets were classified by 4 coders with an overlap of 10% for double coding, that is, coders independently assigned codes to the same set of tweets. The 1000 profile descriptions were categorized into 6 distinct categories with a directed content analysis approach [22] with an additional category “others.” This “others” category was further defined through exploring the profile descriptions, resulting in a total of 12 categories.

The second round aimed to ensure consistency and accuracy between the coders. In total, 100 randomly selected profile descriptions of tweets were allocated to 12 user categories by each coder (Table 2) and agreements, disagreements and potential additions to the categories were discussed. A “bag of words” was compiled for each user category, providing descriptive insights into the characteristics of the users (Table 2 and Multimedia Appendix 2).

**Table 2.** User category and bag of words used for classification.

Index	User category	Profile keywords–“bag of words” (Version 6)
1	Health institute	ICGP^a^; WHO^b^; NHS^c^; public health; ECDC^d^; HSE^e^; HPSC^f^; clinic; hospital
2	Health professional	health worker; GP^g^; consultant; nurse; MD^h^; specialist; physician; clinician; surgeon
3	University/Researcher	university; researcher; PhD; student; scientist; academic professor; academia; principal lecturer
4	Influencer	influencer; blogger; vlogger; coach; YouTube
5	Teacher	teacher; teach; school; education; children
6	Politician	politics; government; governor; cabinet; council; minister; councilor; ambassador; MP;^i^ Secretary of state; Fine Gael; TD^j^; Mayor
7	Sports	football; rugby; run; swim; tennis; exercise; sports club; pickleball
8	Journalist	journalist; report; news; reporter; columnist; reviewer; media; correspondent; editor
9	Charity	charity; church; ngo; foundation; donations
10	Public	community; union; group; nature; lover; adventure; travel; live; life; world; freedom; farm; pet; cat; dog; walks
11	Known personality	views my own, “True” in verified status
12	Artist	artist; actor; actress; music; writer; singer; photography; movie; sing; play; cinema, orchestra

a ICGP: Irish College of General Practitioners.

b WHO: World Health Organization.

c NHS: National Health Service.

d ECDC: European Centre for Disease Prevention and Control.

e HSE: Health Service Executive

f HPSC: Health Protection Surveillance Centre.

g GP: General Practitioner.

h MD: Doctor of Medicine.

i MP: Member of Parliament.

j TD: Teachta Dála.

In the third round, 250 randomly selected profile descriptions were coded by 4 coders and compared with 3 different ML models using the same bag of words: Lbl2TransformerVec model (unsupervised) [23], SGRank model [24], and SetFit model [23].

The ML models classified many influencers as “Public,” which was rectified by allocating influencer to any user with more than 3000 followers. The 3 models’ performance was evaluated using the profile descriptions of 250 tweets (third round of coding) from 4 coders. Majority voting was used to generate a final coding for the 250 profile descriptions and filtering out profiles that had less than 3 coders agreement. A final dataset of 165 profile descriptions was left after this filtering. The 3 models performance was compared with the manual allocation and the final classification was based on the majority allocation.

#### Message Classification

Similar to the user classification, a directed content analysis with abductive coding was applied. A review of literature resulted in the identification of 5 message categories from a study by Gough et al [25], which are humor, shock/disgust, educational/informative, personal stories, and opportunistic. The dataset was divided into 2 subsets—public tweets and nonpublic tweets—to determine which type of messages the public engages with most.

A total of 3 manual coding cycles were applied, starting the allocation of 100 random nonpublic tweets to the five message categories and an “other” category. In addition, 2 additional categories emerged, namely, fear and advocacy.

In Round 2, a total of 5 coders allocated 100 tweets to 7 message categories and discussed agreements and disagreements leading to a refinement of the message categories. Fear-based messaging was combined with shock/disgust, personal stories were combined with personal statements and a “not enough information” category was added. In the final round, 250 tweets were categorised into 7 message categories, which are humor, shock/disgust/fear-based, educational/informative, personal stories/statements, opportunistic, advocacy, and not enough info, and compared to 2 ML models (GPT-3 [OpenAI] and SetFit; Multimedia Appendix 3).

#### Statistical Analysis

For each tweet, engagement was calculated as the sum of likes, replies, retweets, and quoted tweet count divided by the respective user’s follower count [26,27].

For 87% of the tweets, the engagement was zero due to the lack of followers or likes, replies, or quotes (mainly public users). The final dataset excluded this user category resulting in the reduction to 26% zeros. Zero-inflated Poisson and zero-inflated negative binomial models were applied.

### Ethical Considerations

Only publicly available data were extracted for this study. All personal identifiable information was deidentified during the data cleaning process.

## Results

The total number of tweets extracted was 867,485, which reduced to 802,042 after deleting duplicates. Majority of the tweets (729,619) came from the United Kingdom, 72,350 from Ireland, and 73 were without a location.

The follower count for the dataset showed a wide range, from a maximum of 14,065,098 followers to 0 followers, with an average of 4296 followers. Accounts (users) without followers were investigated to identify inactive accounts or bots and 537 users were removed from the dataset.

Public user category tweets (430,760) were removed from the final dataset as they were not a user category of interest. The final dataset included 370,745 tweets.

### Sentiment Analysis

Out of 260 tweets, coders agreed on 247 tweets (this is when 2-3 coders voted the same), which were compared with the accuracy score and *F*_1_-score (weighted) of the 3 ML models. The RoBERTa-base (Model 3) performed the best and was used to assign sentiment to all tweets (Table 3).

Sentiment was negative for 138,379 tweets (37.3%), positive for 84,939 (22.9%), and neutral for 147,427 tweets (39.8%). Positive sentiment tweets had the highest engagement (29.3%), followed by negative (26.7%) and neutral (20.4%; Table 4).

**Table 3.** Accuracy and *F*_1_-score for sentiment models with machine learning models.

Model	Accuracy	*F*_1_-score (weighted)
1–Distilbert-base-uncased-finetuned-sst-2-english	0.68	0.61
2–Finite automata/bertweet-base-sentiment-analysis	0.58	0.61
3–RoBERTa-base	0.74	0.76

**Table 4.** Frequency and engagement for each sentiment category.

Sentiment	Engagement, %	Frequency, %
Positive	29.3	22.9
Negative	26.7	37.3
Neutral	20.4	39.8

### User Classification

Model 3 (assisted) achieved an accuracy score of 0.73 when 4 to 5 coders were in agreement, and achieved an accuracy score of 0.77 when there was at least one match with one coder—192/250 tweets (Table 5).

Health professionals (8%) significantly outnumbered health institutes (1%), while the number of influencers (32%) was more than twice that of journalists (15%) and politicians (13%). Artists (6%) outnumbered individuals associated with sports (2%), and teachers (3%) were one-third in comparison to university/researchers (14%), see Table 6.

Table 7 shows the frequency and percentage of engagement for each user category and provides valuable insights into their impact and effectiveness in engaging audiences. Health professionals have the highest level of engagement (15%), followed by university/researchers (13%) despite a lower frequency (8%). Even though influencers have high frequency (32%) it does not translate into high engagement with influencers having a lower percentage of engagement at 12%.

Journalists and politicians account for 15% and 13%, respectively, with 8% engagement each. Teachers, known personalities. and charities received engagement of 6%, 4%, and 3%, respectively, at lower frequencies. Sports and health Institutes have the lowest engagement each at 1%.

**Table 5.** Accuracy and *F*_1_-score for user classification with machine learning models.

Model	Accuracy	*F*_1_-score (weighted)
1-Lbl2TransformerVec	0.26	0.21
2-SGRank	0.43	0.43
3-SetFit	0.69	0.67
3-SetFit (assisted)	0.73	0.73

**Table 6.** User categories and their frequency.

User categories	Frequency, n	Frequency, %
Influencer	119,711	32.3
Journalist	54,384	14.7
University/Researcher	50,275	13.6
Politician	47,694	12.9
Health professional	28,963	7.8
Artist	21,730	5.9
Known personality	15,095	4.1
Teacher	12,019	3.2
Charity	11,788	3.2
Sports	5659	1.5
Health institute	3427	0.9

**Table 7.** Frequency and engagement for each user category excluding public.

User category	Engagement, %	Frequency, %
Health institute	0.7	0.9
Sports	1.4	1.5
Charity	3.2	3.2
Teacher	5.5	3.2
Known personality	4.1	4.1
Artist	6.1	5.9
Health professional	15.2	7.8
Politician	7.8	12.9
University/Researcher	12.7	13.6
Journalist	8.2	14.7
Influencer	11.5	32.3

### Message Classification

The 2 models’ performance was evaluated using 250 tweets from 5 coders. Majority voting was used to generate a final coding for the 250 tweets, and filtering out tweets that had less than 3 coders agreement. In addition, 11 tweets were removed because they were identified by the coders as “Not enough information” to code. A final dataset of 203 tweets was left after this filtering. SetFit outperformed GPT-3 and this model achieved 80% accuracy when considering a match with at least 1 coder (192/239 tweets; Table 8).

Personal stories/statements have the highest engagement at 27%, but are not the most frequent tweeted category (22%; Table 9). Shock/disgust/fear-based messages (32%) have 21% engagement. Informative/educational messages have high frequencies (33%) and have 16% engagement. Advocacy messages (8%) have 9% engagement and humor and opportunistic messages have engagements of 4% and 0.5%, and low frequencies 5% and 1%, respectively (Table 10).

**Table 8.** Accuracy and *F*_1_-score for user classification with machine learning (ML) models.

	4 and 5 coders agreement		3, 4, or 5 coders agreement	
	Accuracy	*F*_1_-score (weighted)	Accuracy	*F*_1_-score (weighted)
GPT-3	0.60	0.60	0.51	0.49
SetFit	0.74	0.74	0.64	0.62

**Table 9.** Frequency and engagement for each message type.

Message type	Engagement, %	Frequency, %
Opportunistic	0.5	0.7
Humor	3.7	4.7
Advocacy	9.1	8.5
Personal stories/statements	26.7	21.8
Shock/disgust/fear-based	21	31.9
Informative/educational	15.5	32.5

**Table 10.** Message types frequency and engagement percentage.

Message category	Example tweet	Frequency, n	Engagement, %
Informative/ educational	Coronavirus Daily Update: As at 06 Mar 2022, in the Isle of Man there have been 23328 confirmed cases. #coronavirus #iom #coronaupdate	120,317	15.5
Shock/disgust/ fear-based	@Mysturji @AntacsB @Keir_Starmer General strike? We’re already basically on one. Take to the streets? And die of a pandemic?	118,238	21
Personal stories/ statements	Celebrating my end of Self-isolation period with some improvised gluten free macaroni and, er, fussili and cheese. #norecipe #foodie #satisfied @ Dublin, Ireland	80,881	26.7
Advocacy	‚ÄòEach person who has died in this pandemic is a loved person, a life gone too soon and a family torn apart.' Hold a public inquiry into the Government’s handling of the Covid-19 pandemic #Covid19 - Sign the Petition!	31,340	9.1
Humor	The government says we need to exercise social distancing, stay indoors as much as possible and behave like other people might be carrying a disease like this is all new, but I‚Äôve been doing it since about 2002 #introvert #hermit	17,302	3.7
Opportunistic	In light of #pandemic financial challenges for families, I considered the 10% property tax base increase on Waterford #households trying to recover from the pandemic morally wrong in 2020. Yesterday I voted against #LPT 10% increase &amp; my party again!	2667	0.5

### Statistical Analysis

A zero-inflated negative binomial model was applied with informative/educational tweets and neutral sentiment as reference categories. Health professionals received more engagement with advocacy, personal stories/statements and humor. Health Institutes observe higher engagement with advocacy, personal stories/statements and tweets with a positive sentiment (Multimedia Appendix 4).

Journalists and teachers observe higher engagement with advocacy, personal stories/statements and humor while artists have more engagement with shock/disgust/fear-based messages and positive sentiment. Charity organizations, universities, and researchers have more engagement with advocacy and personal stories/statements but not with shock/disgust/fear tweets.

Known personalities have most engagement with advocacy and humor-based, which is opposite for sports entities. Sports entities have more engagement with personal stories/statements and positive sentiment. Politicians and influencers have more engagement with advocacy, personal stories/statements and humor, while they should avoid opportunistic tweets. Table 11 shows the message type and sentiment each user category should tweet and avoid to get maximum engagement.

**Table 11.** Findings of zero-inflated model.

User category	Message type to post	Message type to avoid
Health Institute	Advocacy, personal stories/statements, and positive sentiment	Opportunistic, shock/disgust/fear-based, and negative sentiment
Artist	Shock/disgust/fear-based and positive sentiment	Humor and opportunistic
Charity	Advocacy and personal stories/statements	Humor and negative sentiment
Journalist	Advocacy, personal stories/statements and humor	Shock/disgust/fear-based and negative sentiment
Known personality	Advocacy and humor	Shock/ disgust/fear-based
Teacher	Advocacy, personal stories/statements and humor	Shock/disgust/fear-based and negative sentiment
Health professional	Advocacy and personal stories/statements	Opportunistic and shock/disgust/fear-based
Influencer	Advocacy, personal stories/statements and Humor	Opportunistic and negative sentiment
Sports	Personal stories/statements and positive sentiment	Shock/disgust/fear-based
Politician	Advocacy, personal stories/statements, and humor	Opportunistic and negative sentiment
University/Researcher	Advocacy and personal stories/statements	Shock/disgust/fear-based and negative sentiment

## Discussion

### Principal Findings

This analysis of type, sentiment, and source of COVID-19–related tweets showed what type of tweets have the most engagement for each user group. Overall, personal stories and positive messages have most engagement, even though not for every user group; known persons and influencers have most engagement with humorous tweets. Journalists and teachers were found to have flexibility in their posting strategies, with advocacy, personal stories/statements, and humor being effective for them as well. Artists garnered the most engagement with shock/disgust/fear-based and positive sentiment posts, while charity organizations and universities/researchers had advocacy and personal stories/statements as more engaging message types. Known personalities and sports entities also displayed varying engagement for message types and sentiments, with the former having advocacy and humor-based posts more engaging, while the latter should focus on personal stories/statements and positive sentiment. Politicians and influencers, on the other hand, exhibited higher engagement with advocacy, personal stories/statements, and shock/disgust/fear-based messages, while avoiding opportunistic content.

Positive sentiments in public health messages typically evoke feelings of hope, encouragement, and trust among users, leading to increased sharing behavior [28]. Users are more inclined to engage with and share positive messages that resonate with them emotionally, as they perceive such content as uplifting and supportive [28]. This explains the high engagement received by positive sentiment tweets despite having lower frequency, as shown in Table 4 in this study. Our initial manual categorization ensured a reliable baseline for sentiment analysis in this study. The frequency of negative sentiment tweets and their engagement reflects the widespread anxiety, fear, and uncertainty during the pandemic [29]. The high engagement of tweets with negative sentiments further emphasizes the need to consider emotional content in information dissemination on social media platforms such as Twitter. In this context, future studies should explore the specific impact of tweets by analyzing the responses they generate, including retweets with quotes and replies. This approach would provide additional insights and allow us to evaluate whether tweets achieve their intended impact.

The findings of this study revealed distinct patterns of public engagement across different user categories and message types. Trusted sources are important in shaping public behavior and engagement with health information during crises [30]. Overall, in the case of users, health professionals received high engagement during the pandemic whereas health institutes received the lowest engagement, maybe reflecting their different use of messages. This highlights the importance of using specific message types for each user category to achieve engagement, as recommended by our study. In addition, health experts, such as general practitioners, communicate health information with greater credibility and persuasiveness than nonexperts or institutes [31].

Internet-based health communities and social media platforms influence public health behaviors and engagement levels [32]. The definition and expansion of sources (user categories) in this study provides a framework to develop specific messaging by user group. Our findings reiterate the importance of understanding audience and tailoring engagement strategies based on category-specific behaviors for optimal engagement [33-35].

### Comparison With Previous Work

Most studies have focused on the content of the tweets to understand public reactions or sentiment [36,37], trends during the COVID-19 pandemic [38,39], or vaccinations [40-42].

In one study, Twitter data were explored using machine learning to examine how public opinions and discussions changed throughout the COVID-19 epidemic [36]. Trends in social media discussions during the pandemic were explored using sentiment analysis and topic modeling, which produced useful information about the public discussion around the pandemic [38]. These studies focused mainly on the content of tweets and their engagement but did not explore their association with sources, message type, or sentiments.

The classification of tweets based on types enriched the analysis by capturing the multifaceted nature of communication during the pandemic and provided an understanding of how different message categories influence public engagement and sharing behavior. This study also added to the existing literature by introducing 2 new message categories—advocacy and personal statements—while modifying the existing categories to enable application and provide guidance in framing future public health communications.

Public health literature on message types is very limited. A scoping review on the health risk communication with the public during a pandemic found a lack of studies on the modes of communication [43]. One study discussed the framing of effective COVID-19 messages to connect individuals to authoritative content, emphasising the importance of positive and gain-framed messages [44]. Similarly, personal experiences increased the salience of public health messaging, particularly in promoting sanitation and hygiene practices [45]. Public health messaging during the lockdown in New Zealand showed the importance of consistent messaging principles such as transparency, timeliness, empathy, and clarity [46]. None of these studies used defined message categories and the definition and recommendation of message types for different user categories is a major contribution of our study. This will help content creators, particularly health intervention planners, in choosing the right mix of message and sentiment type to increase their engagement.

A similar study of social media messages explored account type and message structure, taking elements such as hashtags, hyperlinks, mentions, and any images or videos into account but only counting retweets as engagement [8]. They found that tweets with hashtags, videos, and pictures were retweeted more often, while tweets with links had fewer retweets. Furthermore, tweets with sentiment were more frequently retweeted than tweets with neutral sentiments. In our study, the user profile and engagement were explored through engagement metrics such as likes, retweets, reply count, and quote count. We found health institutes to be the least engaged user category, while Xie et al [8] found national health authorities received more engagement when compared with provincial accounts. However, their analysis was limited to the study of the official (national and provincial) public health agencies’ Sina Weibo posts only (a China-only microblogging platform).

This study’s novel approach lies in the examination of engagement for a 2-year period across different user categories for different message types and sentiment, providing insights into public’s response toward different messages and sources. The volume of data and methodology used allows us to provide insights that were not addressed in the existing body of work. This differs from the current studies with similar research objectives, such as a comparative study between Poland and Jordan [47] on social media’s role focused on the disparities in platform choices and message efficacy. Another study categorized COVID-19-related tweets into themes to understand public sentiment. The study identified 5 themes for message categories, which were general information, health information, expressions, humor and others, but used a small dataset [48]. Furthermore, a study examined Canadian public health tweets, revealing that tweets promoting action garnered more engagement than purely informational ones. However, retweets were used as the measure for engagement, unlike our study, where we took into consideration a more comprehensive method for calculating engagement [49]. This is another strength of our study, where we compared engagement across message types and user categories instead of solely depending on likes for comparison.

Our findings suggest a correlation between message type, sentiment, source credibility, and engagement. Our study shows that audience engages with personal stories and positive messages the most. Also, with varied users, different types of messages yield engagement. Our study provides guidance for social media–based public health campaigns for developing messages for maximum engagement.

### Limitations

We included just 3 factors (sentiment, user type, and message type) for the analysis, which was leading to variance in our analysis. Other factors not recorded or captured may also influence engagement, for instance, time or day of posts, hashtags, images, etc. For our study, we also excluded tweets from the public user category, which was almost 50% of the dataset as it was not required to address this study’s research questions.

### Conclusion

Our study provides a framework to develop social media messages according to sentiment and message type for different users. Health professionals and institutes and other users can build on the results to improve effective communication through social media channels.

## Supplementary material

10.2196/59687 Multimedia Appendix 1 Sentiment analysis.

10.2196/59687 Multimedia Appendix 2 User classification.

10.2196/59687 Multimedia Appendix 3 Message classification.

10.2196/59687 Multimedia Appendix 4 Results of zero-inflated model.

10.2196/59687 Checklist 1 STROBE checklist for cross-sectional studies.

## Abbreviations

API: application programming interface
ML: machine learning
STROBE: Strengthening the Reporting of Observational Studies in Epidemiology
